# Annotations

**Published:** 1895-06-08

**Authors:** 


### ANNOTATIONS.

### Should Measles be Notified?

THERE are medical men who assert that notification of infectious diseases is a disappointing proceeding, and that, taking it all round, the "game is hardly worth the candle." On the other hand, many persons consider notification and isolation a perfect "panacea" for epidemics. Manifestly, therefore, it is desirable to get hold of every scrap of trustworthy evidence which can be secured, and to make it as extensively available for medical practitioners and other sanitarians as possible. Dr. Sydney Marsden, D.P.H., Medical Officer of Health for Birkenhead, sends us a summarisation of twelve years' experience of the notification of measles in that town. Admitting the value of notification of many other epidemic diseases, he argues this special case: Is it worth while to notify measles? To this question many practitioners unhesitatingly answer "No." Dr. Marsden, however, sums up the results of his twelve years' experience in these words: "Our experience of the notification of measles is that the advantages derived from such notification fully justify the continuance of it." What are those advantages? Dr. Marsden thinks they consist in epidemics checked and death-rates lowered. If this be so, then obviously notification must be continued. But other medical officers of health do not report epidemics checked and death-rates lowered. What is the reason of the difference between the experience of Dr. Sydney Marsden and that of some other health officers? The secret of Dr. Marsden's success lies, he considers, in early notification. Get prompt information about your first cases, he says, in effect; then isolate immediately; shut up schools at once; cleanse and disinfect houses thoroughly; a single day of prompt action may check an epidemic. We cannot but express our sympathy with Dr. Marsden rather than with those medical officers of health who pin their faith to dilatoriness and *laisser faire*, but it must be remembered that notification, and even removal to hospital, does not operate for good merely by the isolation of the cases dealt with. The matter is far too complex to be explained in so simple a manner.

### Alcohol in Workhouses.

TEMPERANCE writers continue to be jubilant over the steady decrease in the amount of alcohol used in workhouses, a diminution of practically 60 per cent. being stated to have taken place between 1871 and 1891, the amount used having fallen in that time from a value of £82,554 to £34,332. As a fact, the diminution is considerably greater than is expressed by these figures, for in the interval the number of the inmates who are not able-bodied has very considerably increased, and it is to them that alcohol is chiefly, if not exclusively given. We cannot, however, entirely join with those who would enforce upon the sick and aged in workhouses any special restriction of diet which is not generally accepted among a similar class outside. So long as the broad ground is taken that alcohol is a luxury which the guardians are not justified in providing for the paupers, the matter can be argued on genera principles along with the questions of tea, tobacco, better food, raiment, and accommodation, which are now agitating the poor-law world. We doubt, however, whether this is the reason which leads temperance advocates to urge the abolition of the use of alcohol in workhouses. It is because they consider the use of alcohol wrong and unjustifiable on moral grounds that they wish it to be stopped. When those who are free to choose for themselves, decide by their conduct that alcohol is a bad thing, and when it ceases to be used in common life, then by all means let it cease in workhouses; but while the guardians and those who elect them are constant in their practical admission of the dietetic virtues of alcohol we would have none of such vicarious temperance, and would leave the medical officer as free a hand in the prescribing of alcohol as in the ordering of any other detail of the workhouse diet. It must be recognised, whenever this question of the treatment of paupers is discussed, that the inmates of workhouses are mostly feeble folk, and that a large number of them are ill in such degree that, but for the workhouses, they would be in hospitals. Under such circumstances we doubt the propriety of putting pressure on medical officers to adopt methods of treatment for their paupers different from what they adopt in the treatment of their patients outside. While those who are free to choose take alcohol in their diet, and while doctors, in cases where everything is at their disposal so that they will but cure their patients, still prescribe it, we ought to hesitate to fetter them in the treatment of our paupers, or to take pride in such a very vicarious form of virtue as the lessening of the consumption of alcohol in workhouses, except so far as it concerns the able-bodied.

### Our Hospital Sunday Supplement.

SUNDAY, June 16th next, is the day set apart as Hospital Sunday in the Metropolis. Year by year the sympathy with the careful and useful work of the Fund grows. This was never more forcibly illustrated than by the recent announcement of two sums of nearly £50,000 for investment through this fund for the permanent benefit of the Metropolitan hospitals. We have collected many interesting and new facts, which will be published in a Special Supplement of twenty-eight pages, full of graphic illustrations, with which will be presented a fine picture entitled, "Just Before Bed Time," being an incident taken from the daily life of sick children as presented in Queen Victoria Ward at the London Hospital, Whitechapel. The demand for copies of this supplement is already unprecedented, upwards of thirty of the largest churches having already applied for a large number of copies to present to members of the congregations present at the services on Sunday next, the 9th inst. Hospital Sunday is the opportunity for the multitude to give generously to the hospitals, and our Special Illustrated Supplement will, we hope, enable everybody to grasp the facts. It should help the preachers to bring home these facts to the minds and hearts of thousands of persons who have hitherto taken little or no part in the succour of the sick poor of London, who constitute a vast multitude of suffering humanity.

